# The Synthesis of BODIPY-TKI Conjugates and Investigation of Their Ability to Target the Epidermal Growth Factor Receptor

**DOI:** 10.3390/targets1010005

**Published:** 2023-08-30

**Authors:** Simran Dhingra, Prajesh Shrestha, Arpan Chowdhury, Zehua Zhou, Seetharama D. Jois, Maria da Graça H. Vicente

**Affiliations:** 1 Department of Chemistry, Louisiana State University, Baton Rouge, LA 70803, USA; 2 Department of Pathobiological Sciences, School of Veterinary Medicine, Louisiana State University, Baton Rouge, LA 70803, USA; 3 School of Basic Pharmaceutical and Toxicological Sciences, College of Pharmacy, University of Louisiana at Monroe, Monroe, LA 71201, USA

**Keywords:** BODIPY, TKI, EGFR binding, SPR, erlotinib, computational modeling, microscopy

## Abstract

A near-IR BODIPY was covalently conjugated via its isothiocyanate groups to one or two Erlotinib molecules, a known tyrosine kinase inhibitor (TKI), via triethylene glycol spacers, to produce two novel BODIPY-monoTKI and BODIPY-diTKI conjugates. The ability of these conjugates to target the intracellular domain of the epidermal growth factor receptor (EGFR) was investigated using molecular modeling, surface plasma resonance (SPR), EGFR kinase binding assay, time-dependent cellular uptake, and fluorescence microscopy. While both the BODIPY-monoTKI and the BODIPY-diTKI conjugates were shown to bind to the EGFR kinase by SPR and accumulated more efficiently within human HEp2 cells that over-express EGFR than BODIPY alone, only the BODIPY-monoTKI exhibited kinase inhibition activity. This is due to the high hydrophobic character and aggregation behavior of the BODIPY-diTKI in aqueous solutions, as shown by fluorescence quenching. Furthermore, the competition of the two Erlotinibs in the diTKI conjugate for the active site of the kinase, as suggested by computational modeling, might lead to a decrease in binding relative to the monoTKI conjugate. Nevertheless, the efficient cellular uptake and intracellular localization of both conjugates with no observed cytotoxicity suggest that both could be used as near-IR fluorescent markers for cells that over-express EGFR.

## 1. Introduction

Cancer is second most leading cause of death for both men and women [1]. For example, non-small-cell lung cancer (NSCLC) accounts for 81% of cases with a 5-year relative survival rate of only 23%. The majority of NSCLC cases are diagnosed in patients 65 years old or older when the cancer has already reached an advanced and/or metastatic stage [2]. Therefore, the early-stage diagnosis of tumors using non-invasive molecular imaging techniques has become crucial in cancer treatment. Current imaging techniques employed for early detection of lung cancer include chest X-ray (CXR), computed tomography (CT), and positron emission tomography (PET); however, these have some drawbacks, such as exposure of patients to ionizing radiation, relatively low spatial resolution, and high cost, limiting their applications [3]. Therefore, there is a need to develop alternative methods that allow for the early detection of tumor cells by selectively targeting over-expressed receptors at their surface and facilitating treatment planning.

Stanley Cohen and Rita Levi-Montalcini received the 1986 Nobel Prize in physiology for their discovery of the epidermal growth factor receptor (EGFR) [4]. These cell surface receptors belong to the family of receptor tyrosine kinases (RTK) and comprise three major domains: the extracellular domain (ECD) bearing ~620 amino acids and a ligand binding site; the transmembrane domain (TMD); and a cytoplasmic intracellular domain bearing ~540 amino acids that consists of the tyrosine kinase domain (TKD) and a carboxyterminal tail (CTT) with ~230 amino acids [5,6]. The EGFR is activated by the binding of various high-affinity ligands, such as EGF, TGF-α at the ECD of the receptor. Upon activation, EGFR goes from its inactive monomeric form to an active homo/heterodimeric form. EGFR dimerization induces intracellular protein-tyrosine kinase activity leading to autophosphorylation of several tyrosine residues in the C-terminal domain; this elicits downstream activation and initiates several signal transduction cascades that lead to DNA synthesis, cell proliferation, cell migration, adhesion, apoptosis, and angiogenesis, among others. Changes to the EGFR gene (overexpression of receptor, dysregulation or mutation, amplified expression of EGFR binding ligands), however, can lead to continual or abnormal activation of the receptors, causing unregulated cell division that causes various types of cancers. Therefore, tumor cells that over-express EGFR can be targeted for early detection of cancers by combining a fluorophore with molecules that display specific binding for EGFR [7].

Alfred Treibs and Franz-Heinrich Kreuzer were the first to report 4,4-difluoro-4-bora-3a,4a-diaza-s-indacene dyes, commonly known as boron dipyrromethenes or BODIPYs. For the last three decades, BODIPYs have remained the focus of considerable research due to their highly desirable properties for various applications, including strong absorption peaks with large molar absorption coefficients (ε = 50,000–100,000 M^−1^cm^−1^), high fluorescence quantum yields with sharp fluorescence emissions, and relatively long excited singlet state lifetimes (>5 ns) [8]. Additionally, BODIPY dyes show remarkable stability under physiological conditions and are not readily affected by changes in polarity or the pH of their environment. BODIPYs are electron-rich systems with a highly versatile core structure, thereby allowing easy derivatizations to modulate their stability, solubility, and spectroscopic properties.

While the optical and the near-infrared (NIR) imaging range is between 400–2500 nm, the absorbance by various tissue components such as water, melanin, proteins, and hemoglobin (Hb) between 200–650 nm, as well as tissue autofluorescence, can lead to high background fluorescence. Additionally, tissues can reflect, refract, and scatter the incident photons. Therefore, the wavelength range 650–1450 nm is considered the “therapeutic diagnostic window”, with the least tissue absorption and maximum tissue penetration. BODIPY dyes can be carefully designed to both absorb and emit light within the imaging window for applications in bioimaging [9,10].

Two predominant therapeutic approaches are currently employed in clinical investigations against increased EGFR signaling. These approaches consist of the use of monoclonal antibodies (mAbs) directed at the ECD of EGFR and the use of small molecule tyrosine kinase inhibitors (TKIs) that target the catalytic tyrosine kinase domain [11]. TKIs directly inhibit the tyrosine kinase autophosphorylation by binding to the ATP-binding pocket of the receptor. TKIs owe their ability to inhibit the kinase activity to competitive binding with ATP, making them good candidates for targeted therapy against cells over-expressing EGFR [12]. In general, TKIs bind reversibly and/or irreversibly and are divided into first-, second-, third-, and fourth-generation TKIs, depending on their site and mode of action. First-generation inhibitors include the FDA-approved Gefitinib and Erlotinib; these bind reversibly to the ATP binding site of EGFR. The second-generation inhibitors bind irreversibly (covalently), and the third- and fourth-generation TKIs are currently under investigation.

BODIPY dyes have been conjugated with various drugs, vitamins, hormones, lipids, and carbohydrates [13] but have never been conjugated with TKIs. Zheng et al. reported the conjugation of 4-anilinoquinazolines, derivatives of the TKI Gefitinib, with an organoruthenium complex through an amide linkage [14]. This complex showed enhanced inhibitory potential against EGFR over-expressing breast cancer cells without compromising the Ru(II) center reactivity toward the DNA model compound 9-ethylguanine [14]. Both Ru(II)- and Ru(III)-based compounds have shown promising antitumor activities [15]. Bourkoula et al. reported the synthesis of Re and ^99m^Tc complexes with the TKI derivative 6-amino-4-bromophenyl)amino)quinazoline [16]. Biological studies revealed that both the Re and ^99m^Tc complexes bind reversibly to the catalytic kinase domain of EGFR and directly inhibit EGFR autophosphorylation, thereby inhibiting the growth of A431 cells that over-express EGFR [16]. Ji et al. have developed a novel series of Ru complexes with 4-anilinoquinazoline derivatives of Gefitinib [17]. The Ru(III) complex with 4-(3^′^-chloro-4^′^-fluoroanilino)-6-(2-(2-aminoethyl)aminoethoxy)-7-methoxyquinazoline showed higher inhibitory potency against MCF-7 cells, and early-stage apoptosis, than Gefitinib [17].

Recently, two novel Ru(II) conjugates were synthesized with aminoquinazoline derivatives in 1:1 and 2:1 stoichiometric ratios linked via a triethylene glycol spacer [18]. The Ru(II) conjugate containing the (4-bromophenyl)-aminoquinazoline proved to be the most promising inhibitory agent against grade IV malignant glioma cells [18]. More recently, Cheruku et al. reported higher tumor cell accumulation, improved long-term tumor control, and fast clearance from the normal tissues upon conjugation of Erlotinib with a chlorophyll derivative at different positions around the tetrapyrrole ring [19]. Based on the above reports and our observation of efficient cellular accumulation of BODIPY conjugates [8,10], we envisioned the conjugation of one or two TKI Erlotinib molecules to a near-IR BODIPY fluorophore, functionalized with two isothiocyanate groups, to investigate their ability for targeting and labeling the EGFR kinase domain using cellular assays and computer modeling.

## 2. Materials and Methods

### 2.1. Synthesis and Characterization

#### 2.1.1. General Information

All the solvents and chemical reagents were purchased from Sigma-Aldrich or VWR (St. Louis, MO, USA). Dry solvents were obtained from a solvent purification system, Pure Solv PS-400 (Innovative Technology, Carouge Switzerland). Thin-layer chromatography (TLC) was performed on polyester-backed 0.2 mm silica G plates (Sorbent Technologies, Norcross, GA, USA), column chromatography was performed on 60 Å, 40–63 μM silica gel (Sorbent Technologies, Norcross, GA, USA), and preparative alumina neutral TLC plates (20 × 20 cm, 200 μM) from Sorbent Technologies, Norcross, GA, were used for reaction monitoring and purifications. All ^1^H-NMR spectra were collected using a Bruker AV III 400 MHz spectrometer. Samples were dissolved in either deuterated chloroform (CDCl_3_) with chemical shift (δ) ^1^H: 7.27 ppm or DMSO (DMSO-d_6_) with ^1^H: 2.50 ppm, and TMS was used as an internal standard. Coupling constants (J) are reported in Hertz (Hz). Mass spectra were obtained using an Agilent 6210 ESI-TOF mass spectrometer (Agilent Technologies, Santa Clara, CA, USA) at the LSU Department of Chemistry Mass Spectrometry facility. BODIPY **1** was prepared in a total of nine steps, starting from 1-nitrocyclohexene and ethyl-2-isocyanoacetate, as previously reported by our group [8]. Erlotinib **2** was purchased from AK Scientific, Inc. and used without further purification. The azide-terminated triethylene glycol amino linker (azide-PEG3-amine) was purchased from AK Scientific, Inc. (Union City, CA, USA).

#### 2.1.2. Synthesis of Erlotinib-PEG3 4

Erlotinib **2** (0.100 g, 0.254 mmol) was dissolved in DMF (10 mL), followed by addition of azide-PEG3-amine **3** (0.049 g, 0.228 mmol). CuSO_4_·5H_2_O (0.0076 g, 0.030 mmol) was then added, followed by sodium ascorbate (0.0090 g, 0.045 mmol) dissolved in a 1:1 *t*-butanol:water solvent mixture. The reaction mixture was allowed to stir overnight at 40 °C under inert atmosphere. The solvents were removed under reduced pressure, and the resulting residue was purified by column chromatography using neutral alumina grade III and 10% methanol in dichloromethane for elution to yield product **4** as a dark yellow oil (74.62 mg, 80%). HRMS (ESI-TOF) *m*/*z* calculated for C_30_H_41_N_7_O_7_: 611.3140; Found: [M + H]^+^: 612.3154; ^1^H NMR (400 MHz, DMSO-d_6_) δ 9.59 (t, *J* = 44.7 Hz, 1H), 8.53 (s, 1H), 8.32 (s, 1H), 7.73 (d, *J* = 196.5 Hz, 5H), 4.31 (s, 5H), 3.66 (d, *J* = 94.0 Hz, 19H), 2.94 (d, *J* = 19.6 Hz, 6H), 2.52 (s, 2H), 1.98 (s, 1H).

#### 2.1.3. Synthesis of BODIPY-monoTKI 5

BODIPY **1** (0.005 g, 0.007 mmol) was dissolved in dichloromethane (2 mL) before addition of compound **4** (0.004 g, 0.007 mmol). The reaction mixture was wrapped with aluminium foil. Triethylamine (TEA) (0.007 g, 0.071 mmol) was added, and the final mixture was allowed to stir for 2 h at room temperature. The solvents were removed under reduced pressure, followed by purification using neutral alumina preparative TLC and 3% methanol in dichloromethane for elution to obtain conjugate **5** as a bright blue solid in 10% yield (0.5 mg). HRMS (ESI-TOF) *m*/*z* calculated for C_69_H_66_BF_2_N_11_O_9_S_2_: 1305.4657; Found: [M + H]^+^: 1305.4645; ^1^H NMR (400 MHz, DMSO-d_6_) δ 9.92 (s, 1H), 9.54 (s, 1H), 8.52 (s, 1H), 8.46 (d, *J* = 2.4 Hz, 1H), 8.35–8.12 (m, 2H), 8.06 (s, 2H), 7.95–7.31 (m, 12H), 7.30–7.16 (m, 4H), 6.90 (s, 2H), 6.47–6.33 (m, 2H), 4.65 (s, 1H), 4.56 (t, *J* = 5.3 Hz, 2H), 4.37–4.15 (m, 4H), 3.86 (t, *J* = 5.2 Hz, 2H), 3.83–3.69 (m, 8H), 3.68–3.60 (m, 2H), 3.60–3.44 (m, 14H).

#### 2.1.4. Synthesis of BODIPY-diTKI 6

BODIPY **1** (0.005 g, 0.007 mmol) was dissolved in dichloromethane (2 mL) before addition of compound **4** (0.011 g, 0.018 mmol). The reaction mixture was wrapped with aluminium foil. TEA (0.007 g, 0.071 mmol) was added, and the final mixture was allowed to stir overnight at room temperature. The solvents were removed under reduced pressure, followed by purification using alumina preparative TLC and 3% methanol in dichloromethane for elution to obtain conjugate **6** as a bright blue solid in 24% yield (1.2 mg). HRMS (ESI-TOF) *m*/*z* calculated for C_99_H_107_BF_2_N_18_O_16_S_2_: 1916.7724; Found: [M + H]^+^: 1916.7723; ^1^H NMR (400 MHz, DMSO-d_6_) δ 9.95 (s, 1H), 9.55 (d, *J* = 4.3 Hz, 2H), 8.52 (d, *J* = 8.7 Hz, 2H), 8.47 (d, *J* = 5.4 Hz, 2H), 8.28 (s, 2H), 8.06 (s, 2H), 7.94–7.84 (m, 4H), 7.76–7.64 (m, 6H), 7.53 (s, 4H), 7.44 (t, *J* = 7.9 Hz, 2H), 7.27–7.18 (m, 5H), 6.90 (s, 2H), 6.41 (d, *J* = 9.2 Hz, 1H), 4.56 (p, *J* = 6.1, 5.5 Hz, 4H), 4.33–4.23 (m, 10H), 3.86 (t, *J* = 5.2 Hz, 4H), 3.82 (s, 6H), 3.79–3.72 (m, 8H), 3.64 (s, 3H), 3.57–3.47 (m, 18H), 3.36 (t, *J* = 6.9 Hz, 19H).

### 2.2. Spectroscopy Methods

All the UV–Vis absorption spectra were obtained on Varian Cary 50 Bio spectrophotometer (Agilent Technologies, Santa Clara, CA, USA). A PerkinElmer LS55 spectrophotometer was used to record the emission spectra at room temperature. Spectrophotometric grade DMSO was used as solvent for dissolving BODIPY **1** and its bioconjugates **5** and **6**. Quartz cuvettes of 1 cm path length were used. Relative fluorescence quantum yields (Φ_f_) were calculated using methylene blue as the reference using the following equation: Φ_x_ = Φ_st_ × Grad_x_/Grad_st_ × (η_x_/η_st_)^2^, where the Φ_X_ and Φ_ST_ are the quantum yields of the sample and standard, Grad_X_ and Grad_ST_ are the gradients from the plot of integrated fluorescence intensity vs. absorbance, and η represents the refractive index of the solvent (x is for the sample and st for the standard).

Octanol-HEPES Buffer Partition Coefficients (logP)

The partition coefficients (logP) were measured using our previously reported procedure [20]. In summary, 0.5 mL of a conjugate stock solution in DMSO (500 μM) was added to a 15 mL volumetric tube containing 4.0 mL of HEPES buffer (1 M, pH 7.4) and 4.0 mL of 1-octanol. After vortexing for 5 min, the phases were separated and diluted with methanol, and the ratio of their absorbance values was determined.

### 2.3. Molecular Modeling and Docking

For BODIPY-TKI conjugates **5** and **6,** 3D structures of the were generated using Insight II (BIOVIA, San Diego, CA, USA) molecular modeling software. 3D structures were generated and energy minimized with 1000 steps of steepest descent method and 3000 steps of conjugate gradient method. Docking studies were performed as described in our previous publication [8]. Briefly, the 3D structure of kinase domain of EGFR was downloaded from the protein data bank (PDB) [18], and water molecules and ligands were removed. The structure of each BODIPY-TKI conjugate was docked to the kinase domain using Autodock 4.2 [19]. Lamarckian genetic algorithm with 10 million energy evaluations was performed for docking calculations, and the structures with lowest docking energy were used as representative structures. Final docked structures were represented using PyMol software Version 2.5.5 (Schrödinger LLC, Portland, OR, USA). As a control, the structure of Erlotinib was removed from the crystal structure and docked using Autodock.

### 2.4. Surface Plasmon Resonance (SPR) Studies

EGFR kinase domain was obtained from Promega (Madison, WI, USA). SPR studies were conducted using immobilized EGFR kinase on a CM5 sensor chip via standard amine coupling (Cytiva LifeSciences, Marlborough, MA, USA) with a Reichert SR7000DC (Depew, NY, USA). The running buffer used was 1× HBS-EP+ (0.01 M HEPES, 0.15 M NaCl, 3 mM EDTA, 0.005% Tween, pH 7.4) (Cytiva LifeSciences) at a flow rate of 25 μL/min. The analytes were prepared using 5% DMSO in 1× HBS-EP and filtered using a 0.45 μm filter. SPR analysis was performed in the concentration range of 6.25 to 200 μM of each conjugate at 25 °C. Sensograms were analyzed for Kd values using 1:1 Langmuir equation with global fit of graphs.

### 2.5. EGFR Kinase Assay

ADP-Glo^™^ kinase assay kit along with the EGFR kinase enzyme system (Promega) were used. Different concentrations of each BODIPY conjugate were prepared in 1× kinase reaction buffer and added to 384 well plates. A total of 2 μL of 1× kinase reaction buffer containing enzyme (10 μM) was added to all wells, followed by a mixture of 1 μL of 1× kinase reaction buffer containing ATP and 1 μL of substrate. The plate incubated for 1 h at room temperature. A total of 5 μL of ADP-Glo^™^ reagent was added to stop the kinase reaction and the plate was incubated at room temperature for 40 min. 10 μL of kinase detection reagent was added to convert ADP to ATP and introduce luciferase and luciferin to detect ATP. After additional 30 min incubation at room temperature, luminescence was measured. An integration time of 0.25–1 s per well served as a guideline. As a control, one EGFR TKI, Lapatinib (Selleck Chem, Houston, TX, USA) was used.

### 2.6. Cell Studies

All cell culture media and reagents were purchased from Fisher, Invitrogen (Carlsbad, CA, USA). Human cell line HEp2 was purchased from ATCC. The cells were maintained in minimum essential medium (MEM) augmented with 10% fetal bovine serum (FBS) and 1% penicillin-streptomycin (P/S) at 37 °C under 5% CO_2_. A 32 mM stock solution of each BODIPYs **1**, **5**, and **6** was prepared in 96% DMSO and 4% Cremophor-EL. The working solutions were prepared by diluting the 32 mM stock with culture medium.

#### 2.6.1. Dark and Photo Cytotoxicity

Human HEp2 cells were plated in a Corning Costar 96-well plate with 15,000 cells per well and were allowed to grow overnight. The cells were exposed to each BODIPY at increasing concentrations up to 100 μM and then incubated overnight (20–24 h) at 37 °C under 5% CO_2_. The loading medium was then removed, and the cells were washed in triplicate with PBS buffer to remove any residual conjugate. Cells were fed culture medium containing 20% CellTiter Blue (Promega, Madison, WI, USA) and incubated for 4 h at 37 °C under 5% CO_2_. The cell viability was determined by fluorescence intensity at 570/615 nm using a FLUOstar OPTIMA microplate reader (BMG Labtech, Cary, NC, USA). For the phototoxicity experiments, the cells were plated, incubated, and exposed to each BODIPY as described above. After removal of the loading medium, the cells were washed in triplicate with PBS buffer, fed fresh culture medium, and then exposed to light using a 600 W Quartz Tungsten Halogen lamp (Newport Corporation, Irvine, CA, USA). The cells were exposed for 20 min while the plate rested on an Echotherm IC50 chilling/heating plate (Torrey Pines Scientific, Carlsbad, CA, USA) set to 5 °C to maintain ambient temperature. Following light exposure, the cells were incubated for 24 h, and then the media was removed and replaced with 20% Cell Titer Blue in culture medium and incubated for 4 h. Cell viability was determined as described above, and the cytotoxicity is expressed as a percentage of viable cells.

#### 2.6.2. Time-Dependent Cellular Uptake

Human HEp2 cells were plated as described above and incubated overnight. Stock solutions of each BODIPY were diluted to 20 μM with culture medium. The cells were exposed to each conjugate at a final concentration of 10 μM at 0, 1, 2, 4, 8, and 24 h time intervals. The loading medium was then removed, and the cells were washed three times with PBS buffer, then solubilized with 100 μL of 0.25% Triton X-100 (Calbiochem, San Diego, CA, USA) in PBS buffer. CyQUANT cell proliferation assay (Fisher, Invitrogen, Carlsbad, CA, USA) was used for cell quantification. BODIPY concentrations were determined by fluorescence intensity at 640/680 nm (excitation/emission) using a FLUOstar OPTIMA microplate reader. The cellular uptake is expressed in ng of compound per cell.

#### 2.6.3. Microscopy

Human HEp2 cells were plated in a 35 mm tissue culture dish (CellTreat, Pepperell, MA, USA) and incubated overnight. The cells were exposed to BODIPY conjugate **6** at 10 μM concentration and incubated at 37 °C for 6 h. Due to the lower cell accumulation of BODIPY conjugate **5**, the cells were exposed to 10 μM concentration and incubated overnight (approximately 18 h). The following organelle tracers were added: LysoSensor Green 50 nM, MitoTracker Green 250 nM, ER Tracker Blue/white 100 nM, and BODIPY FL C5 Ceramide 50 nM. The organelle tracers were added to the medium, and the cells were incubated concurrently with each conjugate and the tracer for 30 min. The loading media was removed, and the cells were washed with PBS buffer in triplicate. The cells were imaged submerged in PBS buffer. The images were captured using a Leica DM 6B microscope (Leica; Wetzlar, Germany) equipped with a water immersion objective and fitted with standard Texas Red, FITC, DAPI, GFP, YFP, and Cy5 filter cubes (Chroma Technology Corporation, Bellows Falls, VA, USA).

## 3. Results

### 3.1. Synthesis

We have previously reported the total synthesis of near-IR BODIPY **1** [8] bearing two amine-reactive isothiocyanate groups. Briefly, the precursor ethyl-4,5,6,7-tetrahydroisoindole ester was prepared via a Barton–Zard reaction [21] between 1-nitrocyclohexene and ethyl isocyanoacetate in the presence of DBU. After acid-catalyzed condensation with 3,5-dimethoxybenzaldehyde and ester cleavage, the corresponding dipyrromethane was obtained, which underwent decarboxylative iodination and oxidation, followed by boron complexation with BF_3_•OEt_2_ in the presence of TEA, to produce the corresponding 3,5-diiodo BODIPY. A Suzuki–Miyaura [22] coupling reaction using an excess of 4-nitrophenylboronic acid in the presence of K_2_CO_3_ introduced two p-nitrophenyl groups at the 3,5-positions of the BODIPY. After aromatization of the BODIPY core using 2,3-dichloro-5,6-dicyano-1,4-benzoquinone (DDQ) in refluxing toluene, followed by reduction of the nitro groups with Pd/C and hydrazine, and conversion of the resulting amino groups into isothiocyanates using 1,1′-thiocarbonyldi-2(1H)-pyridone, BODIPY **1** was obtained in about 35% yield over the nine steps.

**Scheme 1.**
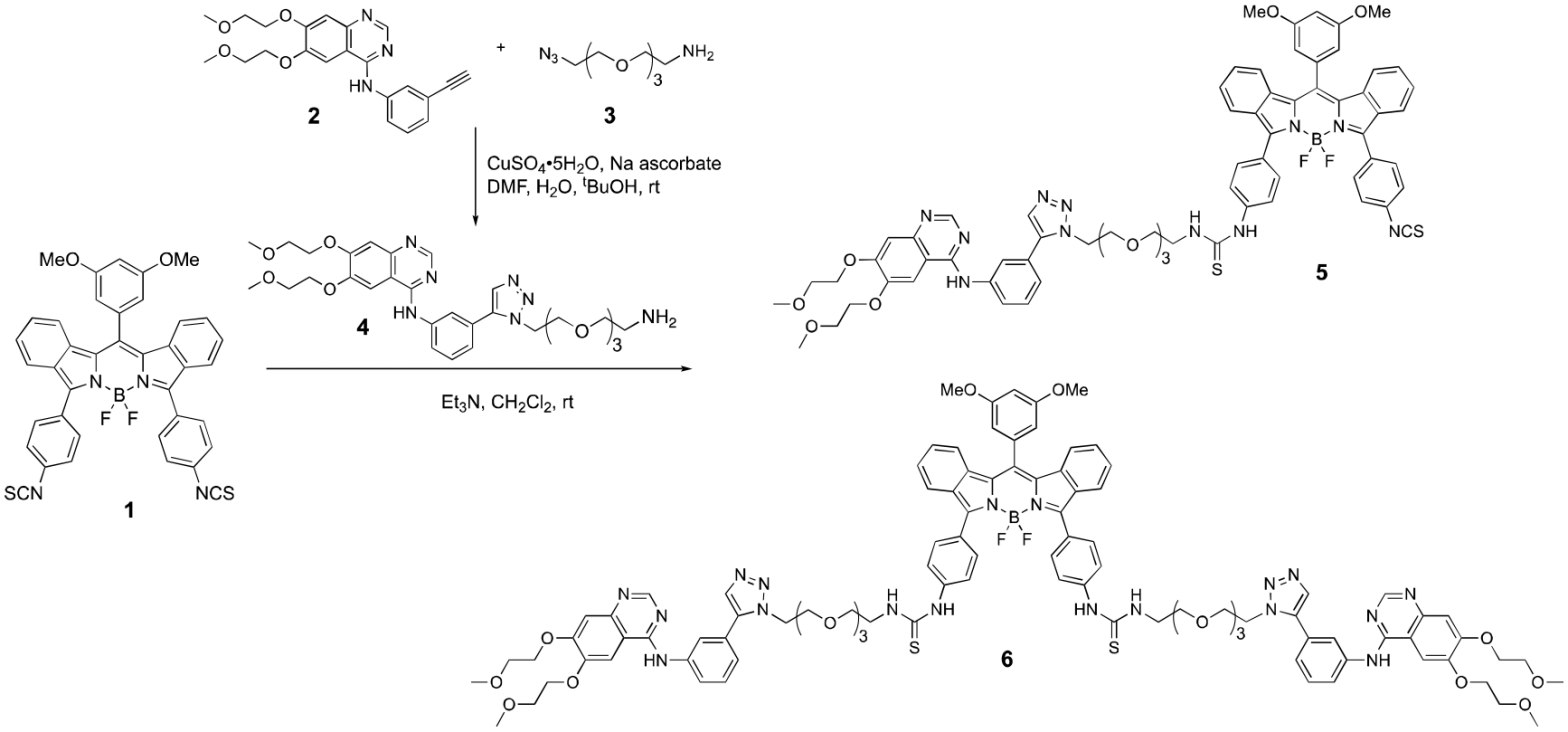
Synthesis of BODIPY-TKI conjugates 5 and 6.

Since Erlotinib contains a terminal acetylene group, a Cu(I)-catalyzed azide-alkyne Huisgen cycloaddition or “click reaction” was used to introduce an amine-terminated triethylene glycol linker suitable for conjugation with BODIPY **1**, as shown in Scheme 1. We have previously reported the use of click reactions for the conjugation of BODIPY fluorophores to carbohydrates and peptides in high yields [10,23,24]. In addition, the conjugation of Erlotinib to various peripheric positions of chlorin and bacteriochlorin macrocycles has been recently reported via a triazole-containing linker [19]. Therefore, the reaction of Erlotinib **2** with commercially available azido-PEG3-amino **3** via a click reaction produced compound **4** in 80% yield (Scheme 1). The amino group of the Erlotinib-linker derivative **4** was then conjugated to BODIPY **1** in the presence of TEA, giving BODIPY-monoTKI **5** as the main product when a 1:1 ratio of reagents was used for 2 h at room temperature in dichloromethane. On the other hand, the BODIPY-diTKI **6** was obtained as the main product when an excess of **4** was used and a longer reaction time (12 h) to achieve reaction completion. The formation of the conjugates was confirmed by HRMS and ^1^H-NMR spectroscopy (see Supplementary Materials Figures S1–S3 and S8–S10).

### 3.2. Spectroscopic Investigations

The spectroscopic properties of BODIPY conjugates **5** and **6**, including absorption and emission wavelengths, Stokes’ shift, and relative fluorescence quantum yields, were investigated in DMSO, and the results are summarized in Table 1 and in Figures S4 and S5 of the Supplemental Materials. As expected, the BODIPY conjugates show strong π → π* absorption and emission bands in the near-IR range, as previously observed [8]. The absorption and emission bands of the BODIPY conjugates **5** and **6** are slightly red-shifted compared with those of BODIPY **1** due to the conversion of the isothiocyanate into a thioamide group. The Stokes’ shifts are within the 34–36 nm range, and the molar absorptivities for conjugates **5** and **6** are slightly lower than for BODIPY **1**, which is in agreement with the previous observations [8]. The fluorescence quantum yields (Φ_f_) were calculated relative to methylene blue in the range of 0.24–0.31 in DMSO. Conjugates **5** and **6** were found to have decreased relative fluorescence quantum yields compared with BODIPY **1** due to the introduction of the Erlotinib-triethylene glycol moieties, which likely increase non-radiative decay.

The calculated polar surface area (PSA) values [25,26], as well as the measured 1-octanol-HEPES buffer partition coefficients (logP) for the conjugates, are also shown in Table 1. Interestingly, the PSA value for BODIPY-diTKI **6** was found to be much larger than that of the monoTKI conjugate **5**, suggesting that the larger diTKI conjugate might have a larger tendency for aggregation. Indeed, the logP determined for the diTKI conjugate is higher than that for the monoTKI conjugate, indicating that the diTKI is more hydrophobic. Both BODIPY conjugates **5** and **6** displayed lower logP values compared with the fluorophore alone, as expected, due to the presence of the ethylene glycol moieties in the conjugates, which decreased their hydrophobic character. The relative aggregation tendency of the two conjugates **5** and **6** in aqueous solution was further investigated by absorption and emission spectrophotometry. A 1% DMSO in HEPES buffer solution of BODIPY-mono-TKI **5** showed a significant decrease in the absorption band intensity, as well as a red-shifted and broadened absorption band, indicating aggregation (see Supplemental Materials Figure S6). In the case of BODIPY-di-TKI **6,** we observed an even more pronounced decrease in the intensity of the absorption band and a complete quenching of the conjugate fluorescence. Furthermore, conjugate **6** showed decreased solubility in DMSO compared with mono-TKI conjugate **5**, as its DMSO solutions slowly became cloudy with time.

### 3.3. Computer Modeling and Docking Studies

**Figure 1.**
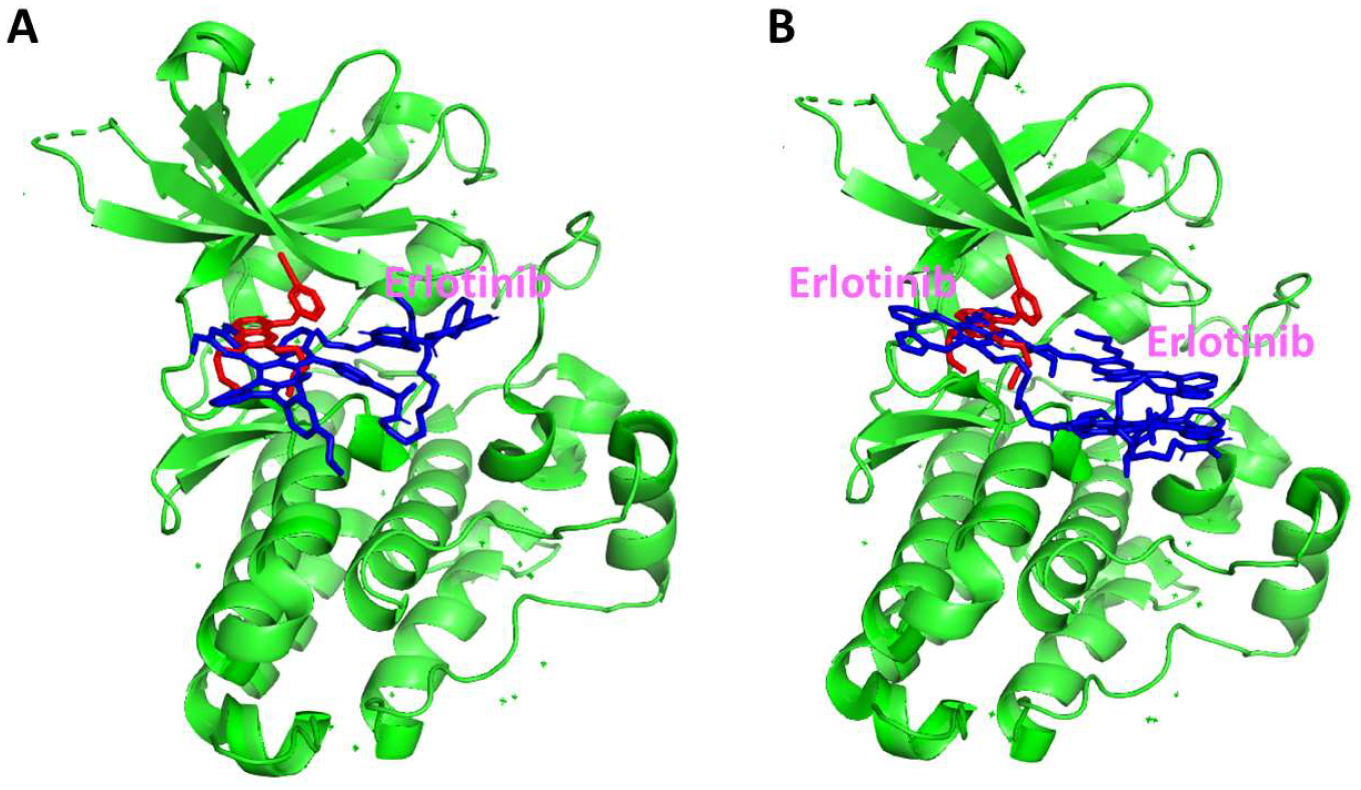
Model of interaction of BODIPY-monoTKI 5 (**A**) and BODIPY-diTKI 6 (**B**) with the kinase domain of the EGFR protein (PDB ID 4hjo) using Autodock. The lowest energy-docked structures are represented. The conjugates are shown in blue, and Erlotinib in the binding pocket is shown in red. The docking energies are −10.22 kcal/mol for 5 and −8.40 kcal/mol for **6**.

To model the binding interaction of the BODIPY-TKI conjugates **5** and **6** with the EGFR kinase active site, a computational docking method was used. First, the TKI was removed from the crystal structure of the EGFR-Erlotinib complex, and Erlotinib was docked to EGFR using Autodock. The lowest energy-docked structure (−9.39 kcal/mol) was docked in the kinase binding site of EGFR. A comparison of the crystal structure of the complex indicated that the docked and crystal structures overlapped at the kinase enzyme activation site (see Supplemental Materials Figure S11). Subsequently, the 3D structures of the BODIPY-TKI conjugates **5** and **6** were generated and docked to the EGFR kinase binding site. When BODIPY-monoTKI **5** was docked to the EGFR kinase, the lowest energy-docked structures had two possible binding modes. In one of them, the BODIPY moiety occupies the Erlotinib position in the crystal structure, and Erlotinib occupies the allosteric site position, as shown in Figure 1A, with a docking energy of −10.22 kcal/mol. In the other docking mode, Erlotinib occupies the kinase active site, and the BODIPY moiety is next to the Erlotinib and the linker, extending outside the kinase active site. In this case, the docking energy was slightly lower, −8.40 kcal/mol. On the other hand, when BODIPY-diTKI **6** was docked to the EGFR kinase, both Erlotinibs in the structures were either near the active site or away from the active site with docking energies of −8 and −7 kcal/mol, respectively. In the lowest energy-docked structure, both Erlotinibs were in the enzyme active site, as shown in Figure 1B. There is a possibility that both Erlotinibs compete for binding to the active site, and hence, the binding of conjugate **6** may not be as efficient as in the case of the monoTKI conjugate **5**.

### 3.4. Surface Plasma Resonance (SPR) Studies

**Figure 2.**
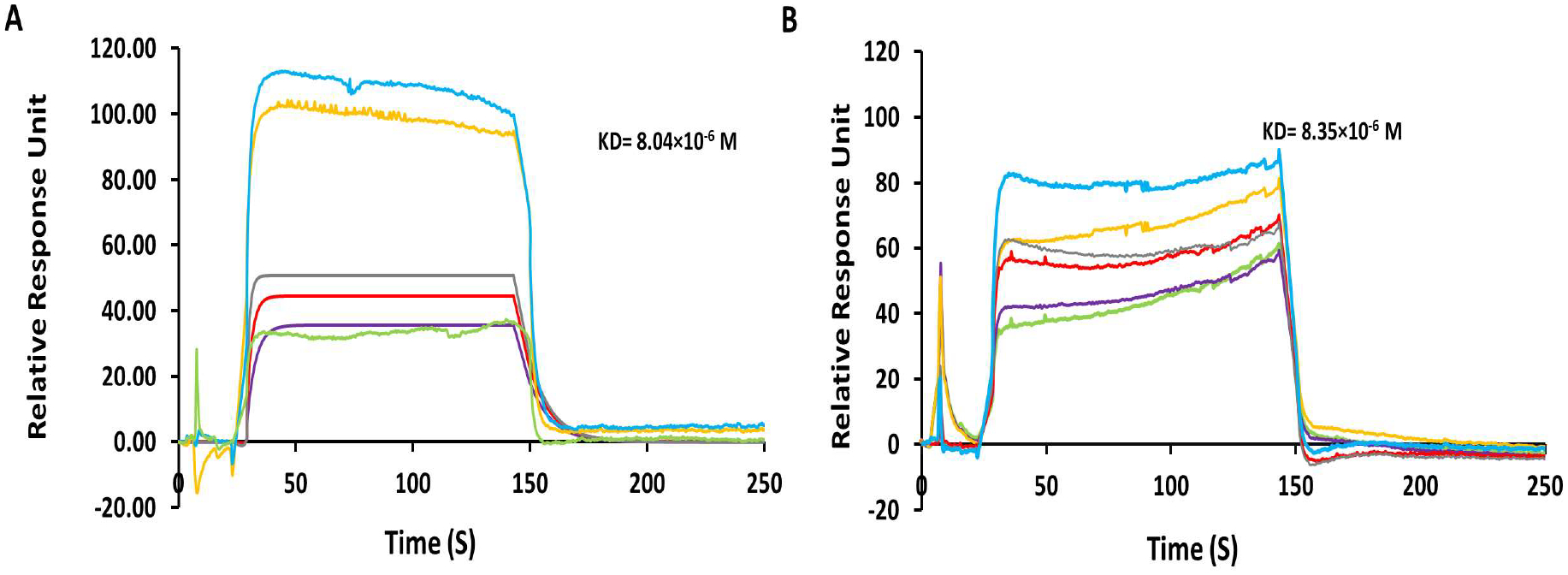
Binding of BODIPY monoTKI **5** (**A**) and of BODIPY diTKI **6** (**B**) to EGFR kinase by SPR analysis. The EGFR kinase was immobilized, and the conjugates were used as the analyte to study kinetics of association and dissociation. Concentrations of the analyte in μM: 200 (aqua), 100 (orange), 50 (gray), 25 (red), 12.5 (purple), 6.25 (green). The SPR sensorgram raw data was used for curve fitting. For final representation, the SPR sensorgrams were processed using Microsoft Excel, and spikes resulting from injections were removed during processing.

The binding of BODIPY-monoTKI **5** and BODIPY-diTKI **6** was evaluated by SPR analysis. The EGFR kinase domain (residues 695 onwards) was immobilized on a CM5 SPR chip. The binding of TKI to the active EGFR kinase was verified by using; the EGFR TKI Lapatinib (see Supplemental Materials Figure S12). When BODIPY-monoTKI **5** wns added to the EGFR kinase on the surface of the chip, a concentration-dependent binding was observed with a retativa response unit of 110 for 200 μM concentration, as seen in Figure 2A. Tho K_*D*_ value obtained from the global fitting of association and dissociation analysis was 8.04 μM. On the other hand, when BODIPY-diTKI **6** was used as the analyte for SPR, binding to EGFR was seen wiah a relative response unit of 80 units for 200 μM concentration. The K_*D*_ vnlue obtained in this case was 8.35 μM (Figure 2B). These results show that both conjugates are able to bind to the EGFR kinase domain.

### 3.5. EGFR Kinase Activity Assay

**Figure 3.**
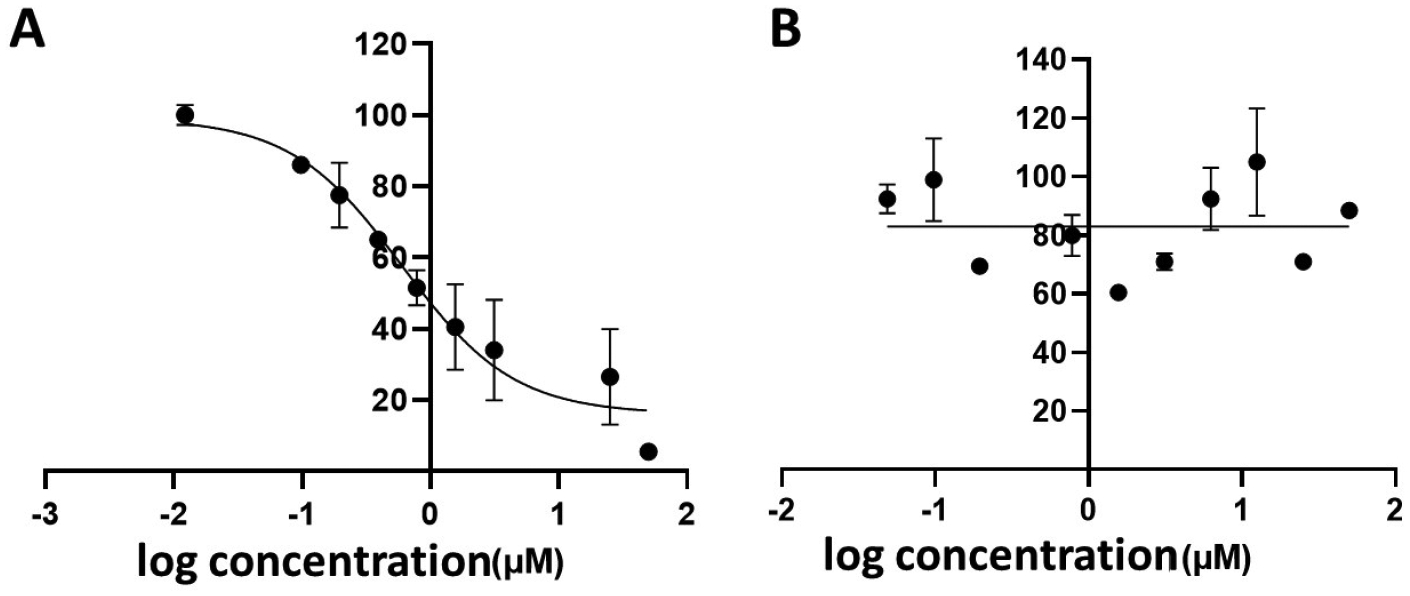
EGFR kinase activity inhibition using a kinase assay. Inhibition of EGFR kinase activity by BODIPY monoTKI **5** (**A**) and BODIPY diTKI **6** (**B**).

The competitive binding of BODIPY-monoTKI **5** and of BODIPY-diTKI **6** to the EGFR enzyme active site was evaluated by a kinase activity assay. In this assay, the ADP kinase reaction is measured by conversion of ADP to ATP. The ATP formed is used in a luciferase reaction that generates luminescence, which correlates with the kinase activity. Therefore, inhibition of the kinase activity is measured by a decrease in luminescence. This assay is often used for screening kinase inhibitors. Therefore, Lapatinib, a well-known TKI [12], was first used in the assay, and a dose-dependent inhibition of the kinase activity was observed (see Supplemental Materials Figure S13). When BODIPY-monoTKI **5** was added to the kinase reaction, a similar dose-dependent decrease in luminescence was observed, as shown in Figure 3A. Therefore, conjugate **5** exhibits kinase inhibition activity, similar to that observed for Lapatinib, suggesting competitive binding of this conjugate to the EGFR kinase binding site. On the other hand, when BODIPY-diTKI **6** was added to the kinase, no dose-dependent response in luminescence was observed, as shown in Figure 3B, suggesting that this conjugate does not competitively bind to the kinase ATP binding site. Nevertheless, SPR analyses indicate that both conjugates can bind to the EGFR kinase, suggesting potentially non-specific binding by the BODIPY-diTKI.

### 3.6. HEp2 Cell Studies

The cytotoxicity, time-dependent uptake, and subcellular localization of the BODIPY-TKI conjugates were evaluated in human HEp2 cells, which show EGFR over-expression. A Cell Titer Blue assay was used to evaluate the cytotoxicity of the conjugates, both in the dark and after exposure to a 1.5 J/cm^2^ light dose. In agreement with previous findings [8], these conjugates showed low cytotoxicity up to 100 μM, as shown in Supplemental Materials Figure S14. The low cytotoxicity of these conjugates is an important property for their potential application as fluorescent bioimaging agents of EGFR over-expressing cells.

**Figure 4.**
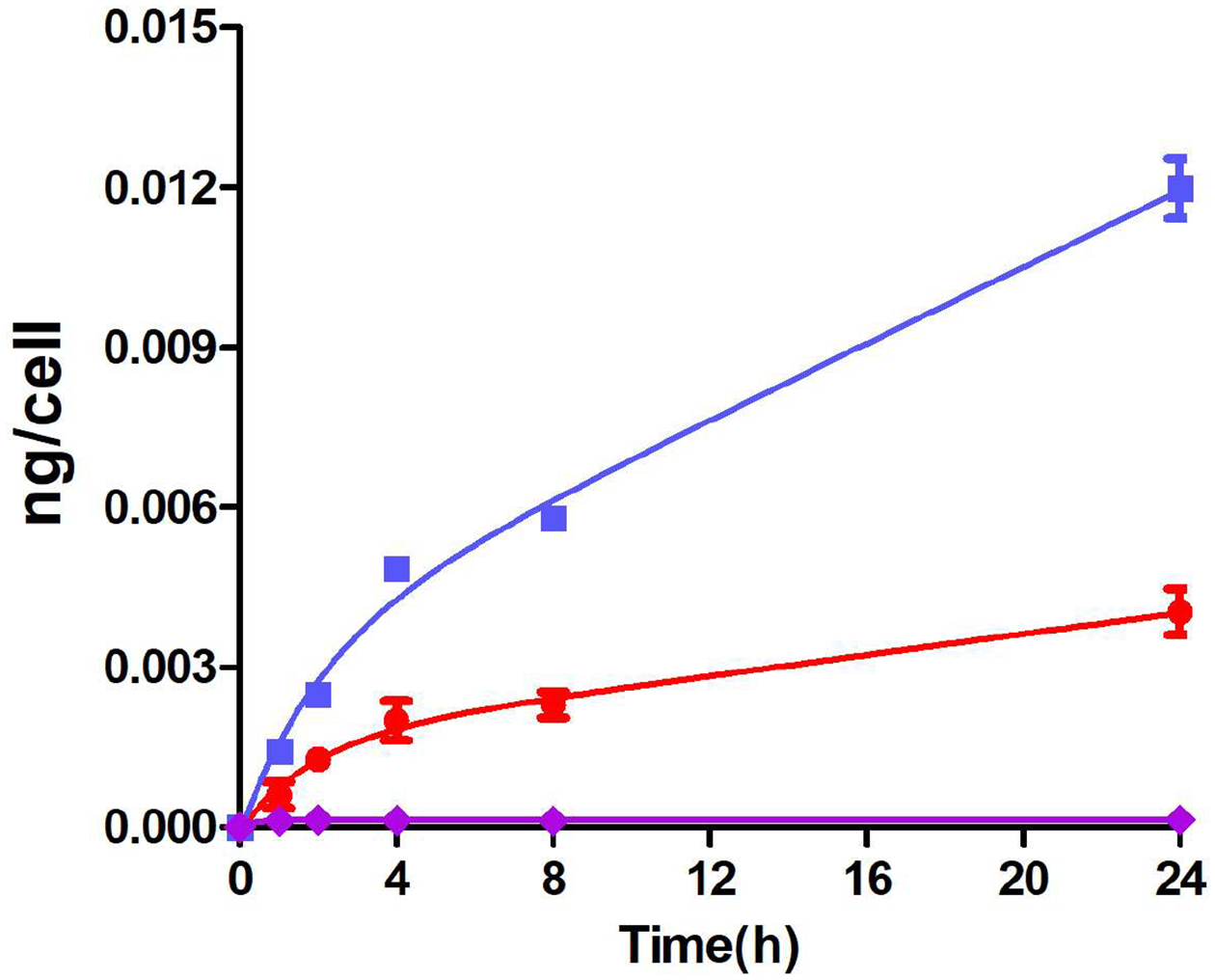
Time-dependent uptake of BODIPY-(NCS)_2_ **1** (purple), BODIPY-monoTKI **5** (red), and BODIPY-diTKI **6** (blue) by human HEp2 cells at a concentration of 10 μM over a 24 h period.

The time-dependent uptake of BODIPY **1**, BODIPY-monoTKI **5**, and BODIPY-diTKI **6** by the HEp2 cells was investigated at a concentration of 10 μM over a 24 h period, and the results are shown in Figure 4. Both conjugates were observed to accumulate efficiently within cells. Interestingly, the BODIPY-diTKI conjugate accumulated much more rapidly within the cells than the BODIPY-monoTKI, and after 24 h, more than twice the amount of the BODIPY-diTKI was found within the cells compared with the mono-TKI conjugate. This is likely due to the increased hydrophobic character of the BODIPY-diTKI conjugate, as indicated by the calculated logP values (see Table 1) and its higher tendency for aggregation in aqueous solutions. Furthermore, our results show that Erlotinib significantly increases the cellular permeability of the BODIPY fluorophore.

**Figure 5.**
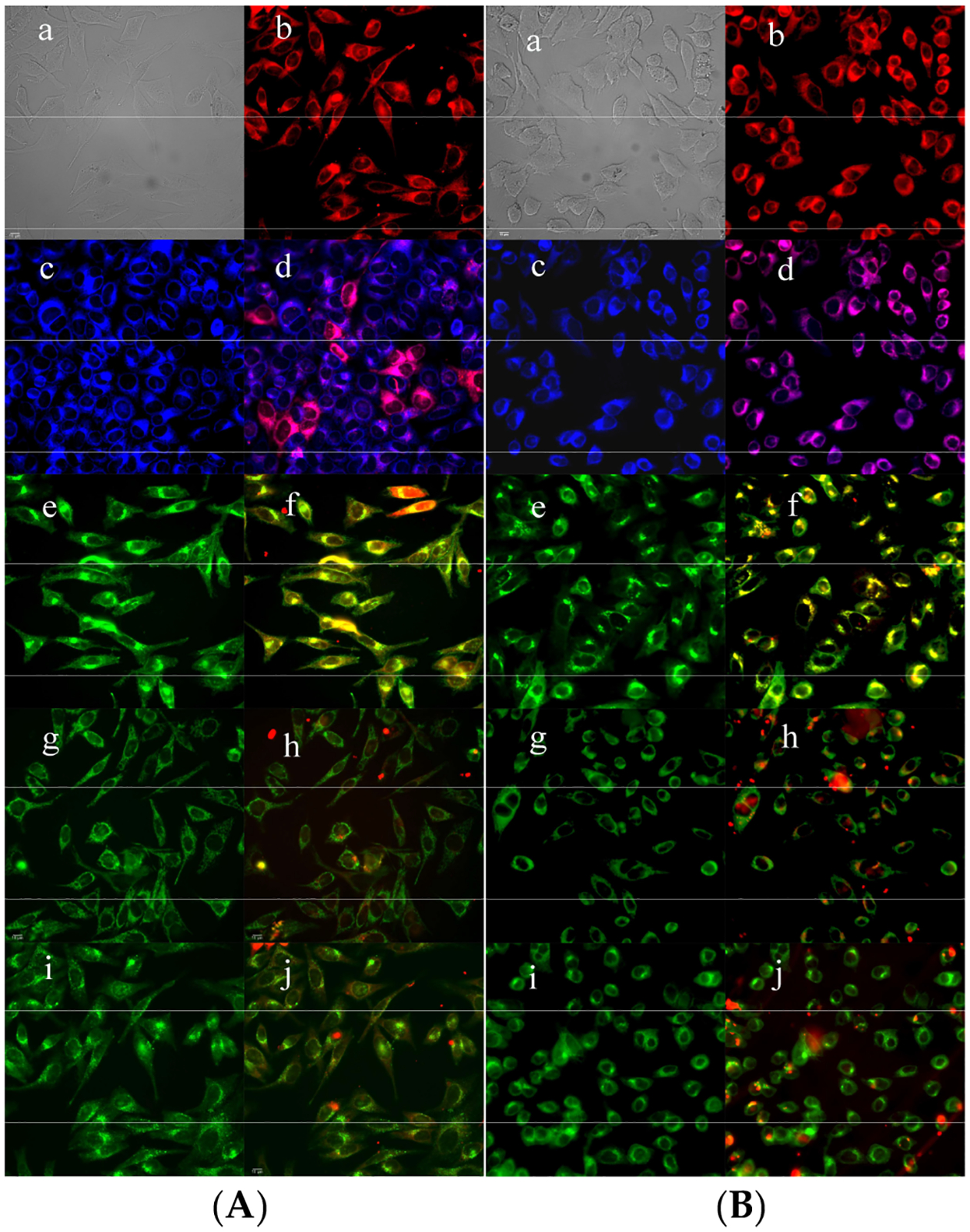
Subcellular localization of BODIPY conjugate **5** (**A**) and **6** (**B**) in HEp2 cells at 10 μM for 6–18 h. (**a**) Phase contrast, (**b**) Fluorescence of BODIPY conjugate, (**c**) ER Tracker Blue/White fluorescence, (**e**) BODIPY Ceramide fluorescence, (**g**) MitoTracker Green fluorescence, (**i**) LysoSensor Green fluorescence, and (**d**,**f**,**h**,**j**) overlays of organelle tracers with BODIPY conjugate fluorescence. Scale bar: 10 μm.

Fluorescence microscopy was performed to further investigate the cell permeability of the conjugates and to evaluate their major and minor sites of intracellular localization. The observed fluorescence signal from each BODIPY conjugate, as seen in Figure 5(Ab,Bb), was mainly intracellular; since the diTKI conjugate accumulated faster within cells, the images were obtained 6 h and 18 h after exposure to conjugate **6** and **5**, respectively. Organelle-specific tracers ER Tracker Blue/White (ER), BODIPY Ceramide (Golgi), MitoTracker Green (mitochondria), and LysoSensor Green (lysosomes) were used for the overlay experiments. Since the BODIPY fluoresces red (Figure 5(Ab,Bb)), the purple color in Figure 5(Ad,Bd) indicates that both conjugates **5** and **6** localize in the ER, and the yellow/orange color in Figure 5(Af,Bf) indicates localization of both conjugates in the Golgi. In addition, conjugate **5** also localizes in mitochondria (Figure 5Ah) to a larger extent than conjugate **6** (Figure 5Bh), and both BODIPY-TKI conjugates were also found to a smaller extent in the lysosomes (Figure 5(Aj,Bj)).

## 4. Discussion

Erlotinib is an FDA-approved first-generation TKI used in the treatment of non-small-cell lung carcinoma (NSCLC), a leading cause of cancer deaths around the world [12]. Since its approval in 2004, the interactions of Erlotinib with the EGFR kinase have been extensively studied to explore the specific interactions between Erlotinib and the amino acid residues in the EGFR kinase binding site [27,28]. It was shown that the quinoline moiety of Erlotinib occupies the adenine pocket of the active kinase, forming multiple hydrophobic and hydrogen interactions, including N1 with Met769 and N3 with Thr766 and Th830. In addition, the quinoline N1 also interacts with the amide nitrogen backbone of Met793. On the other hand, the ethynylphenyl group interacts with the hydrophobic pocket of the EGFR kinase, while the ethoxymethoxy moieties are directed towards the solvent region. The reversible binding of Erlotinib to the ATP binding pocket of the EGFR kinase is known to inhibit phosphorylation at the tyrosine kinase, which blocks signal transduction and inhibits tumor cell growth [12]. Therefore, the conjugation of Erlotinib to fluorophores and photosensitizers is an attractive strategy for the application of the resulting conjugates in the imaging and/or treatment of cancers that over-express EGFR [11,17]. Herein, we describe for the first time the conjugation of Erlotinib TKI to a near-IR BODIPY.

The terminal acetylene group of Erlotinib conveniently allows the use of a click reaction for the introduction of a triethylene glycol linker under mild reaction conditions and in high yield [20,21]. In addition, the resulting triazole group is known to have high photo and chemical stability. Furthermore, the amine-terminated linker readily reacts with the isothiocyanate group(s) in BODIPY **1**, as we have previously observed [8], allowing the preparation of BODIPY-Erlotinib conjugates **5** and **6**, and investigation of their ability for targeting the EGFR tyrosine kinase.

Our results clearly show that both BODIPY-erlotinib conjugates **5** and **6** efficiently internalize within human HEp2 cells that over-express EGFR (up to one order of magnitude greater than BODIPY alone). Interestingly, the di-TKI conjugate accumulates within the cells to a higher extent (approximately 2.5-fold) than the monoTKI conjugate, possibly as a result of its higher hydrophobic character and aggregation that leads to enhanced cellular uptake, likely by phagocytosis. Despite this, we did not observe kinase inhibition using the diTKI conjugate **6**, likely due to its strong tendency for aggregation, as indicated by a drastic quenching of its fluorescence emission in aqueous solution. In contrast, the monoTKI conjugate **5** showed concentration-dependent inhibition of the kinase activity. Nevertheless, SPR studies indicated that both conjugates can bind to the EGFR kinase. Although SPR studies suggest the binding of both the conjugates to the EGFR kinase, they do not specify where the conjugates bind on the kinase domain. The kinase active domain has allosteric sites that are hydrophobic in addition to the ATP binding site. Hydrophobic molecules, such as the diTKI conjugate **6**, can bind to the allosteric site without inducing a dose-dependent inhibition of ATP binding. Since ATP can still bind to the kinase active site, the direct inhibition of the kinase activity by this conjugate was not observed experimentally. Furthermore, docking studies showed that the BODIPY moiety can bind near the kinase active site. In addition, the docking studies suggest that the two Erlotinibs in conjugate **6** can competitively bind to the kinase active site. With the relatively long triethylene glycol linker, the two Erlotinibs compete for binding to the kinase active site, thereby decreasing the binding affinity of the overall diTKI conjugate, as observed in the kinase binding assay. Since it is the quinoline moiety that is essential for binding to the kinase active site, there is a possibility that the two Erlotinibs undergo π-π stacking with each other, preventing the quinoline moiety from binding to the ATP active site in case of the diTKI conjugate **6**. Hence, the kinase assay results showed no dose-dependent inhibition of kinase activity when conjugate **6** was added. Nevertheless, this conjugate accumulated rapidly and efficiently within cells and readily localized intracellularly, mainly in the Golgi, ER, and lysosomes, 6 h after exposure to cells, as shown by fluorescence microscopy. Similarly, conjugate **5** was found to localize intracellularly, mainly in the Golgi, ER, mitochondria, and lysosomes, 18 h after exposure to cells.

## 5. Conclusions

A near-IR BODIPY bearing two isothiocyanate groups was covalently linked to one or two TKI Erlotinib molecules via a triethylene glycol spacer. Either the BODIPY-monoTKI **5** or the BODIPY-diTKI **6** could be obtained as the main product, depending on the conjugation reaction conditions. Using Autodock, it was revealed that both the conjugates show specific binding towards the kinase domain of EGFR with negative binding energies, and SPR studies indicated the binding of both conjugates **5** and **6** to the kinase domain of the EGFR protein. Furthermore, both conjugates efficiently accumulated within HEp2 cells, which over-express EGFR up to one order of magnitude greater than the BODIPY alone. However, using a kinase binding assay, only the BODIPY-monoTKI was observed to inhibit the kinase activity, similar to that observed for the binding of TKI alone. This is likely due to the high hydrophobic character of the diTKI conjugate and its strong tendency for aggregation in aqueous solutions. Our results show that both the BODIPY conjugates **5** and **6** have the ability to bind to EGFR, efficiently internalize within cells that over-express EGFR, and therefore can potentially find application as imaging tools for early detection of EGFR over-expressing tumors.

## Supplementary Material

Supporting Information

**Supplementary Materials:** The following supporting information can be downloaded at https://www.mdpi.com/article/10.3390/targets1010005/s1. Figures S1–S3: ^1^H NMR of compounds **4**, **5,** and **6**, respectively; Figure S4: Normalized UV–Vis absorption spectra of BODIPYs **1**, **5,** and **6** in DMSO; Figure S5: Normalized fluorescence emission spectra of BODIPYs **1**, **5,** and **6** in DMSO; Figure S6: UV–Vis absorption spectra of conjugates **5** and **6** in 1% DMSO/HEPES; Figure S7: Emission spectra of conjugates **5** and **6** in % DMSO/HEPES; Figures S8–S10: High-resolution mass spectrum (ESI-TOF) of compounds **4**, **5** and **6**, respectively; Figure S11: Low energy docked structure of Erlotinib overlapped with the crystal structure of Erlotinib EGFR kinase complex (PD ID: 4hjo). Red sticks, crystal structure of Erlotinib, blue, docked structure of Erlotinib; Figure S12: SPR analysis of binding of Lapatinib a TKI to EGFR kinase domain is used as a positive control to evaluate the binding conjugates to EGFR kinase. Lapatinib is a kinase inhibitor of EGFR/HER2; Figure S13: Inhibition of EGFR kinase activity assessed by kinase activity kit. Lapatinib is a known EGFR/HER2 kinase activity inhibitor; Figure S14: Dark and photocytoxicity of conjugate **6** in human HEp2 cells using a Cell Titer Blue assay.

## Figures and Tables

**Table 1.** Spectroscopic properties of BODIPY-TKI conjugates in DMSO, their calculated polar surface area (PSA), and partition coefficients in octanol-HEPES buffer (logP).

BODIPY	λ_abs_ (nm)	λ_em_ (nm)	Stokes Shift (nm)	Φ_f_^a^	ε (M^−1^ cm^−1^)	PSA (Å^2^)	logP
**1** ^b^	650	686	36	0.35	75,610	124.65	1.41
**5**	656	692	36	0.31	40,200	203.2	1.25
**6**	656	690	34	0.24	53,350	532.11	1.30

a Calculated using methylene blue (Φ_f_ = 0.035) in ethanol at λ_exc_ = 652 nm as the standard.

b Previous work from this laboratory [8].
