# Supplementary material for: The Synthesis of BODIPY-TKI Conjugates and Investigation of Their Ability to Target the Epidermal Growth Factor Receptor
Source: Targets (Basel). Author manuscript; Available in PMC 2026 Jan 16. (PMC12807552; doi:10.3390/targets1010005)
Supplement: Supporting Information [file NIHMS2127673-supplement-Supporting_Information.pdf]

Supplemental Materials

Synthesis of BODIPY-TKI conjugates and investigation of their ability for targeting the Epidermal Growth Factor Receptor

Simran Dhingra [1], Prajesh Shrestha [2,3], Arpan Chowdhury [2,3] , Zehua Zhou[1], Seetharama D. Jois [2,3],*, and Maria da Graça H. Vicente [1,*]

1 Department of Chemistry, Louisiana State University, Baton Rouge, LA, 70803, USA
2 Department of Pathobiological Sciences, School of Veterinary Medicine, Louisiana State University, Baton Rouge, LA, 70803, USA
3 School of Basic Pharmaceutical and Toxicological Sciences, College of Pharmacy, University of Louisiana at Monroe, Monroe, LA 71201, USA
* Correspondence: sjois@lsu.edu; vicente@lsu.edu; Tel.: +1-225-578-7405; Fax: +1-225-578-3458

TABLE OF CONTENTS

Figures S1-S3: ^{1}H NMR Spectra....................S2

Figures S4-S7: Spectroscopic Data....................S3

Figures S8-S10: HRMS Data S6

Figure S11: Docked structure of erlotinib overlapped with the crystal structure of EGFR................S7

Figure S12: SPR of lapatinib binding to EGFR....................S7

Figure S13: Lapatinib inhibition of EGFR Lapatinib....................S8

Figure S14: Cytotoxicity in human HEp2 cells....................S8

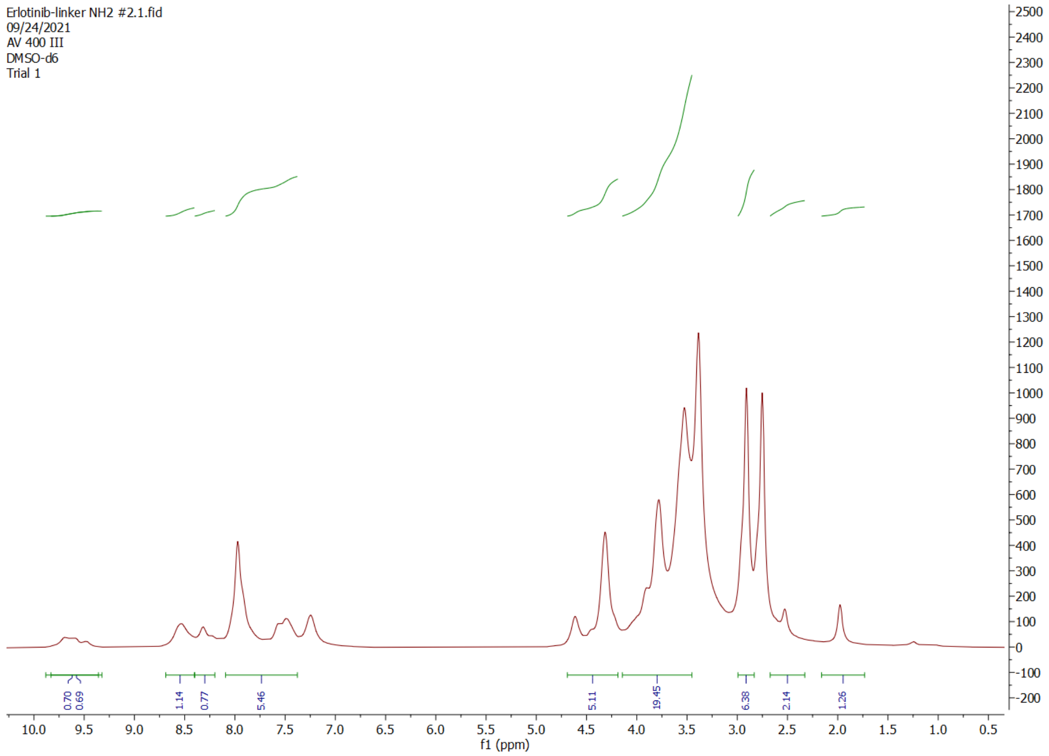

Figure S1. ^{1}H NMR of compound **4**

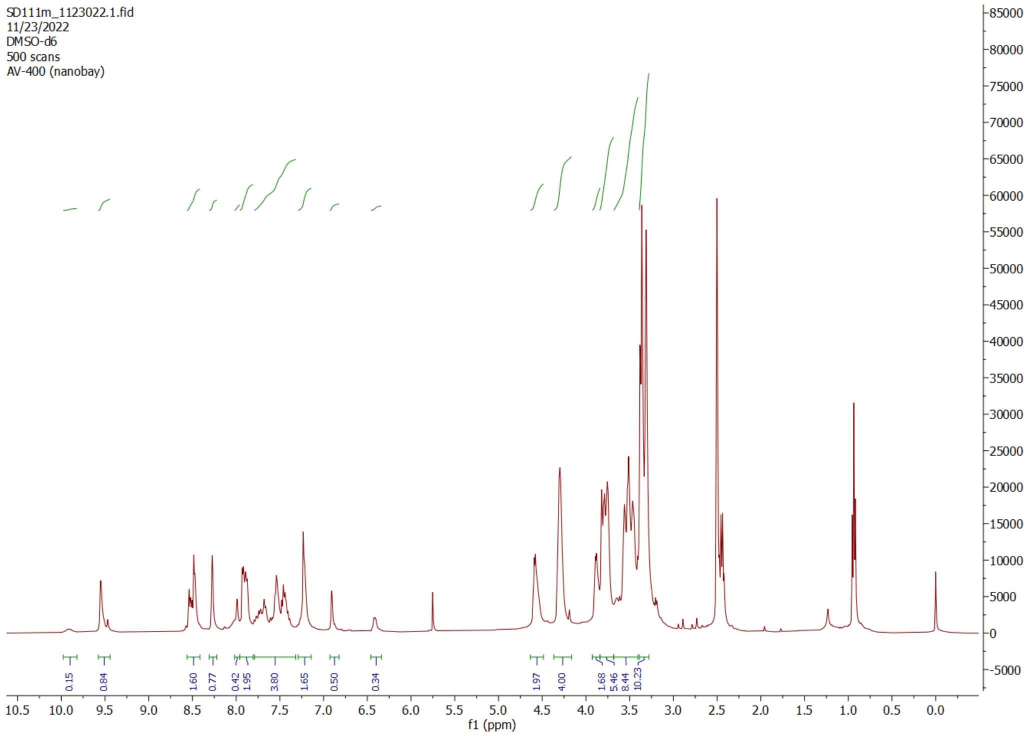

Figure S2. ^{1}H NMR of conjugate **5**

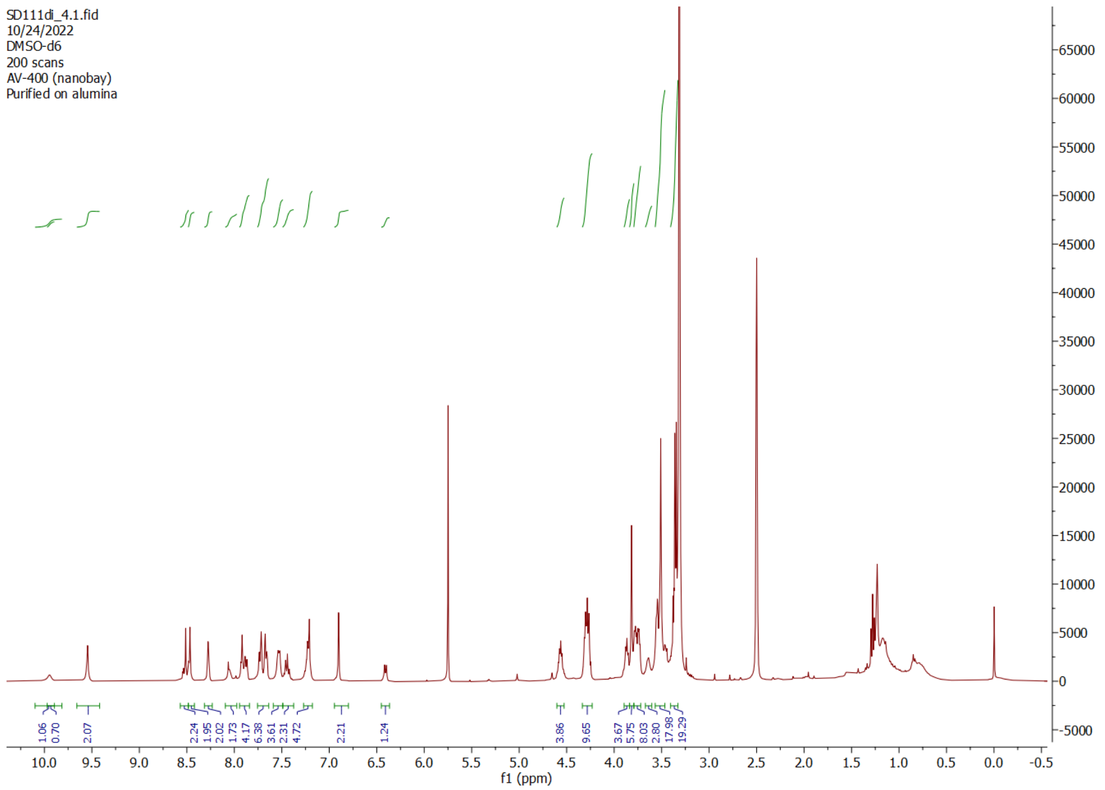

Figure S3. ^{1}H NMR of conjugate **6**

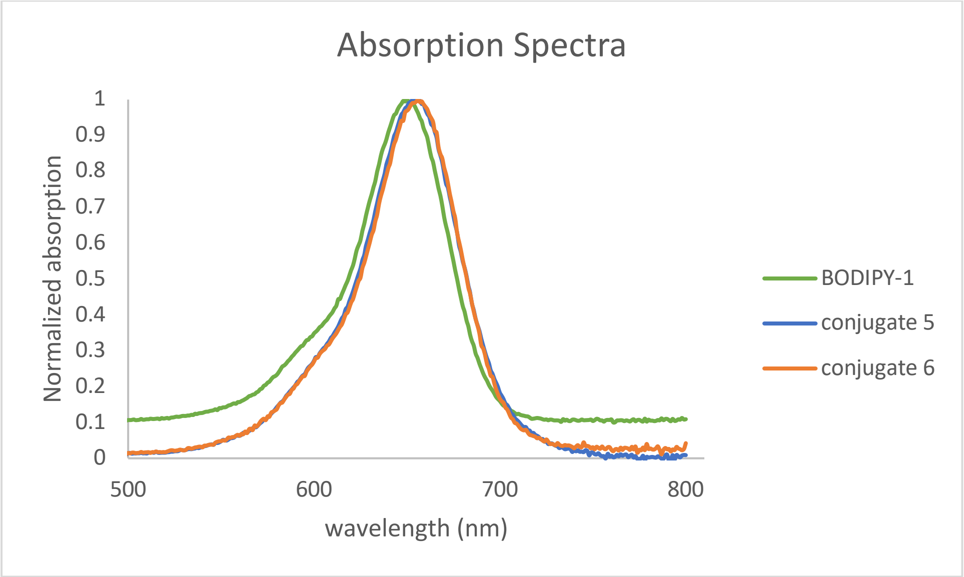

Figure S4. Normalized UV-Vis absorption spectra of BODIPY **1** and conjugates **5, 6** in DMSO

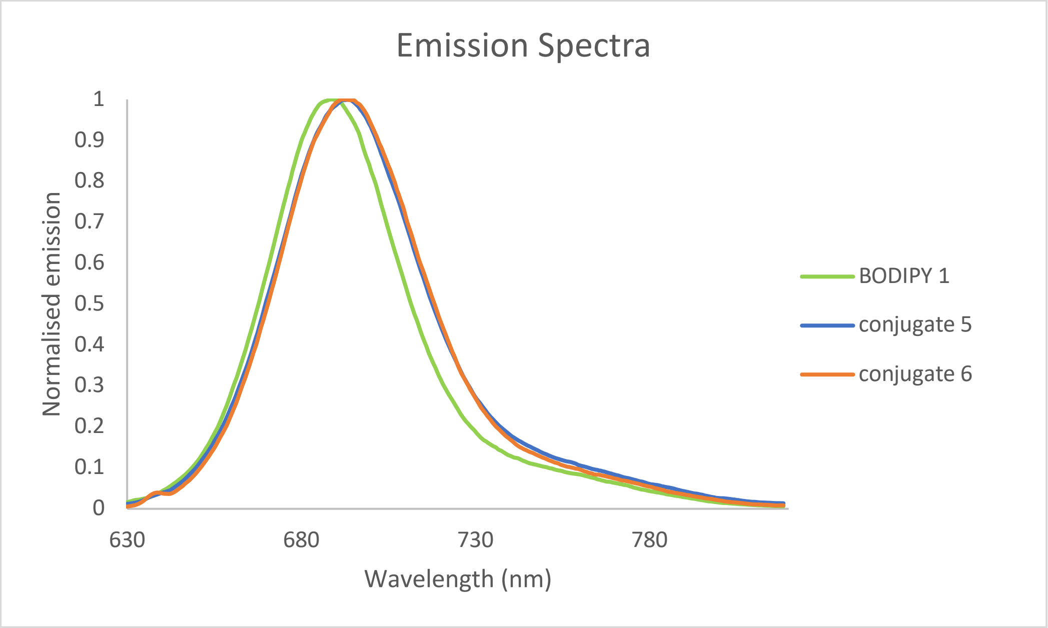

Figure S5. Normalized fluorescence emission spectra of BODIPY **1** and conjugates **5, 6**

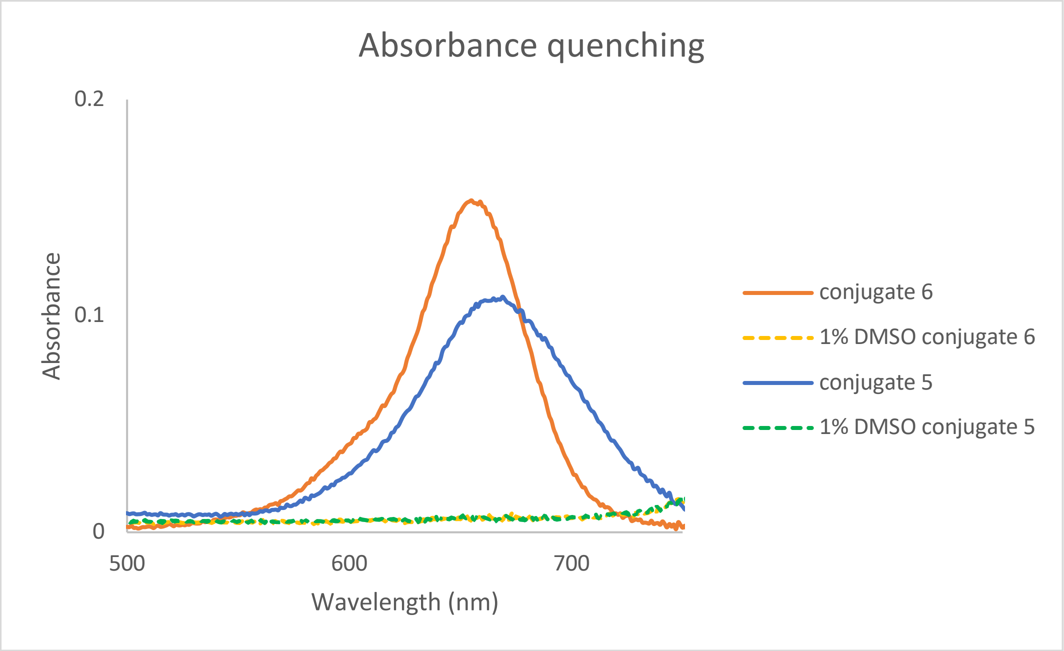

Figure S6. UV-Vis spectra of conjugates **5** and **6** at 1 × 10^{-5}M in a) DMSO solution (solid line), b) freshly prepared HEPES buffer solution containing 1% DMSO solution (dashed line)

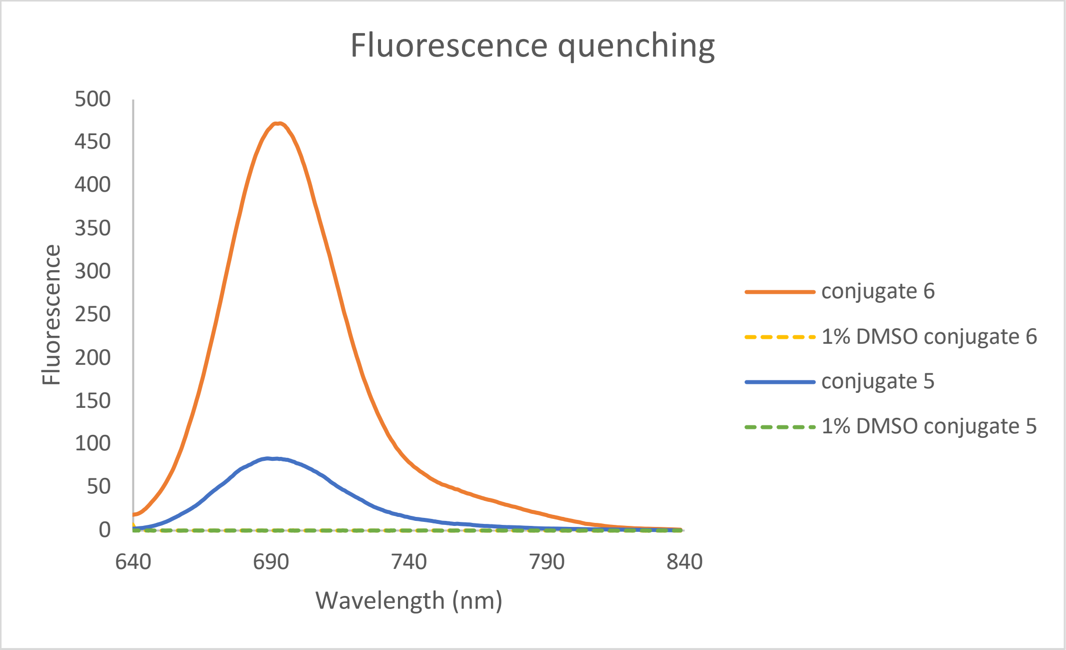

Figure S7. Fluorescence emission spectra of conjugates **5** and **6** at 1×10^{-5}M in a) DMSO solution (solid line), b) freshly prepared HEPES buffer solution containing 1% DMSO solution (dashed line)

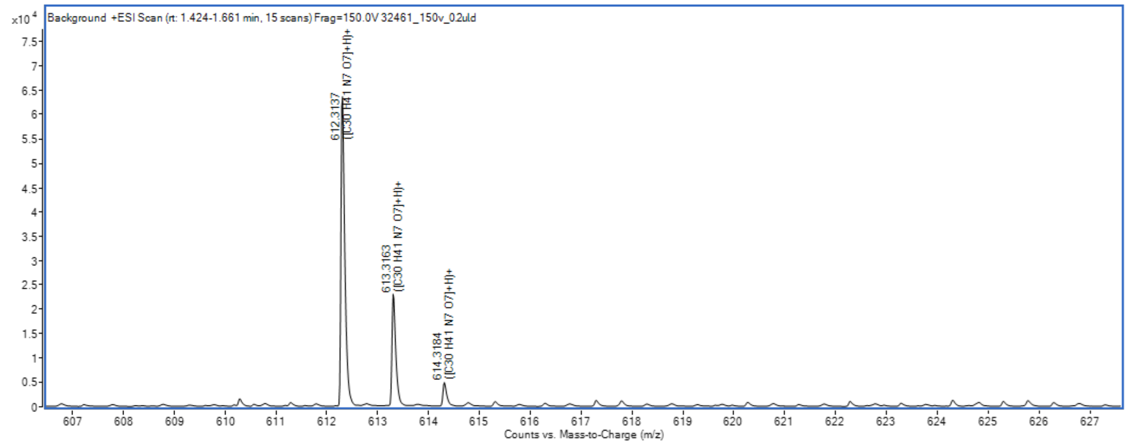

Figure S8. High-resolution mass spectrum (ESI-TOF) of compound **4**

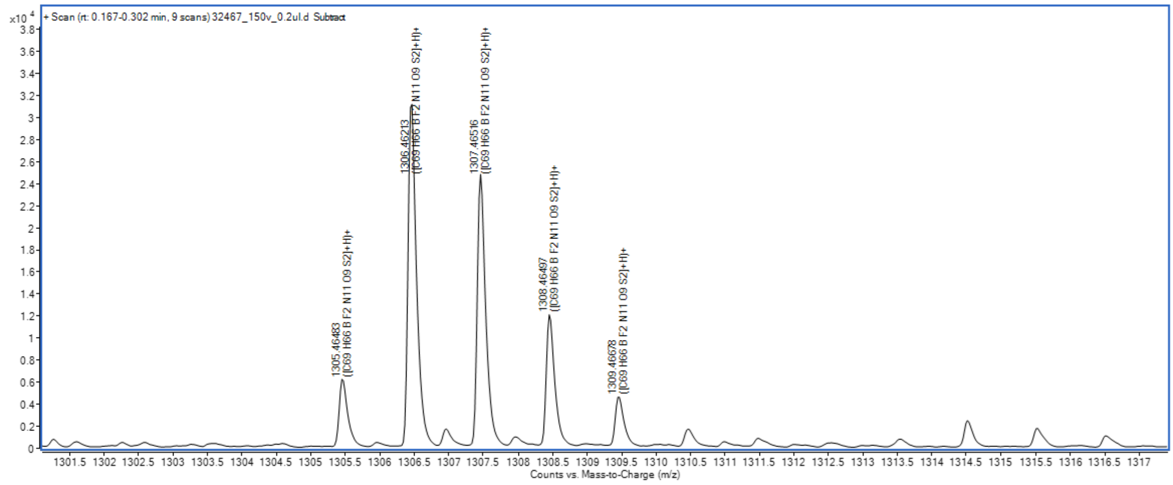

Figure S9. High-resolution mass spectrum (ESI-TOF) of conjugate **5**

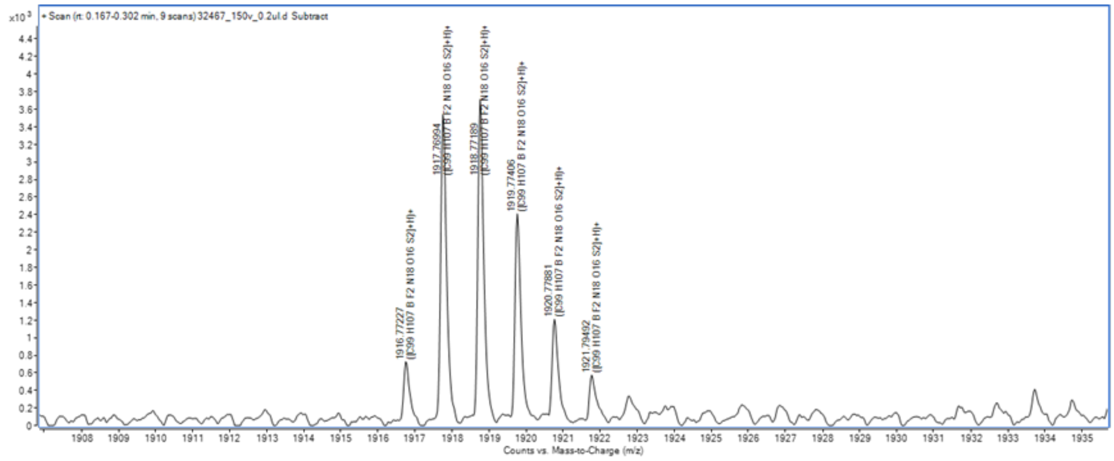

Figure S10. High-resolution mass spectrum (ESI-TOF) of conjugate **6**

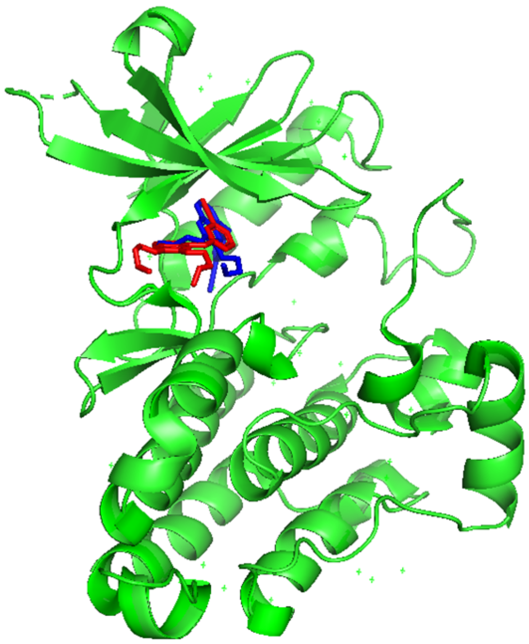

Figure S11. Low energy docked structure of erlotinib overlapped with the crystal structure of erlotinib EGFR kinase complex (PD ID: 4hjo). Red sticks, crystal structure of erlotinib, blue, docked structure of erlotinib.

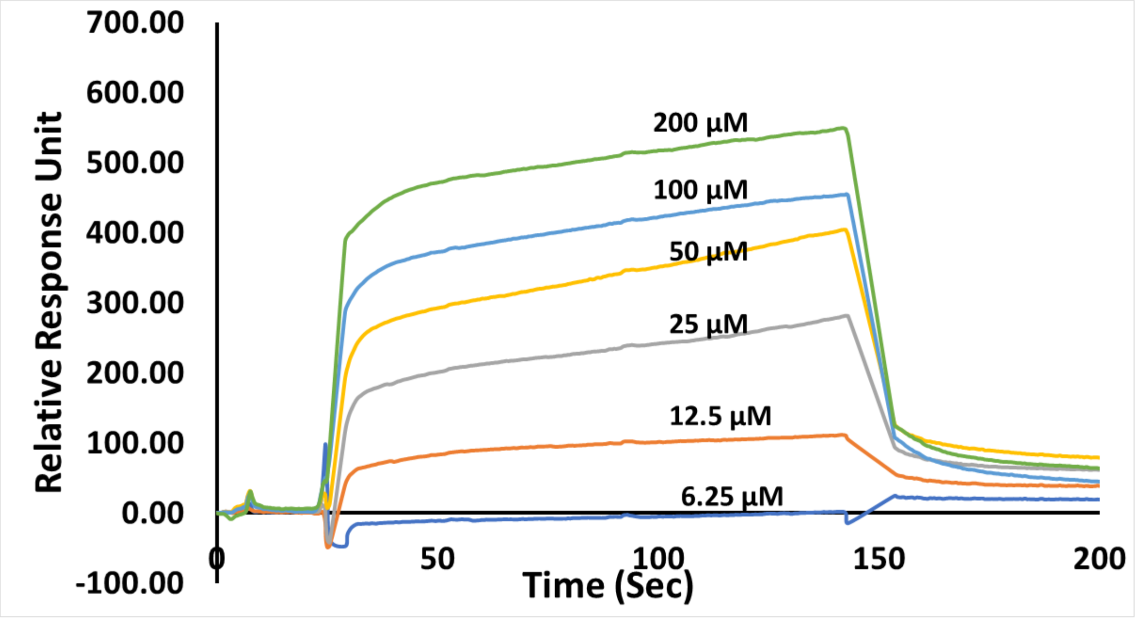

Figure S12. SPR analysis of binding of lapatinib a TKI to EGFR kinase domain is used as a positive control to evaluate the binding conjugates to EGFR kinase. Lapatinib is a kinase inhibitor of EGFR/HER2.

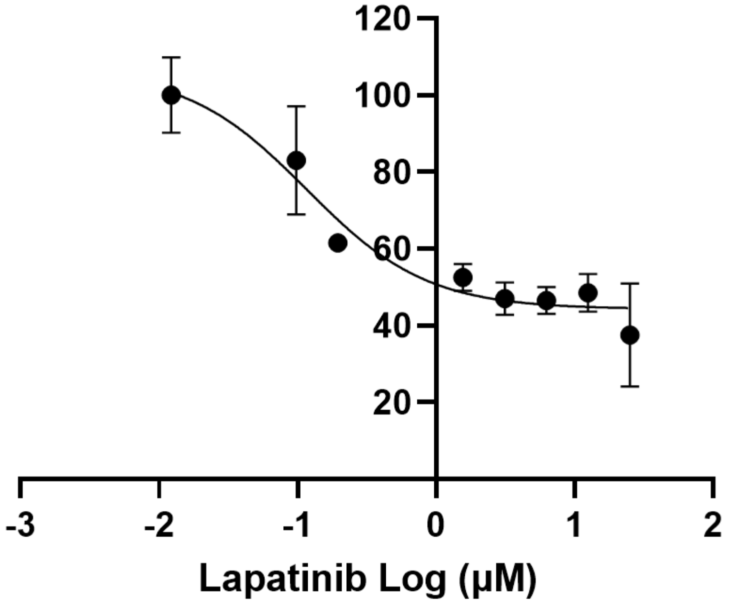

Figure S13. Inhibition of EGFR kinase activity assessed by kinase activity kit. Lapatinib is a known EGFR/HER2 kinase activity inhibitor. Dose dependent inhibition of kinase activity was observed.

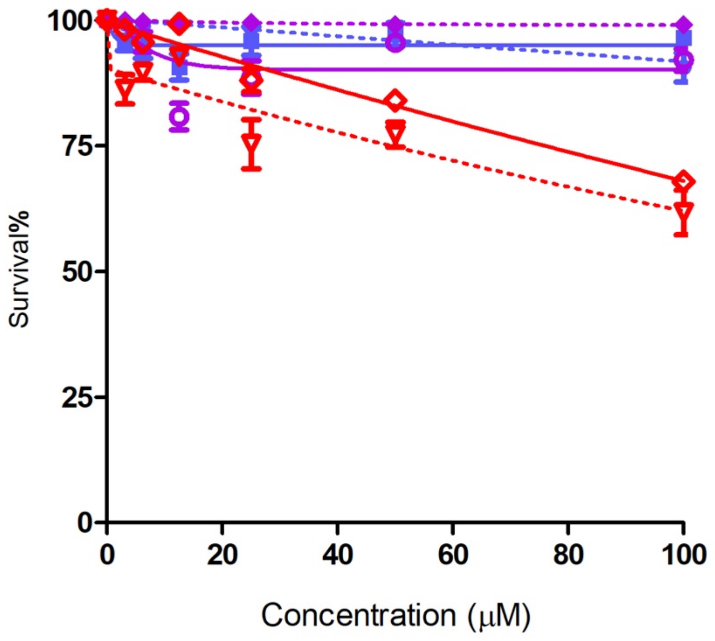

Figure S14. Dark (full line) and photocytoxicity (1.5 J/cm^2, dashed line) of BODIPY 1 (purple), conjugate **5** (red), and conjugate **6** (blue) in human HEp2 cells using a Cell Titer Blue assay.
